# P02.70. Feasibility and effect of chair massage offered to nurses during working hours on stress related symptoms: a pilot study

**DOI:** 10.1186/1472-6882-12-S1-P126

**Published:** 2012-06-12

**Authors:** D Engen, B Bauer, A Vincent, C Luedtke, L Loehrer, S Cha, T Chon, L Dion, N Rodgers, D Wahner-Roedler

**Affiliations:** 1 Mayo Clinic, Rochester, USA

## Purpose

To assess the feasibility and effect of chair massage offered to hospital nurses during working hours on stress related symptoms.

## Methods

Single arm study performed between 10/15/2010 and 12/24/2010 at an academic medical center. A mass e-mail was sent to all nurses working in an inpatient psychiatric and an outpatient pain rehabilitation unit. The first 40 respondents were enrolled; two were excluded due to missing enrollment data. A 15 minute chair massage once a week for 10 weeks was provided by one of three Certified Massage Therapists available 3 days a week. Instruments used included the Perceived Stress Scale (PSS-14), Smith Anxiety Scale (SAS), and Linear Analogue Scale Assessment (LASA) scale. Mean and standard deviations of PSS-14, SAS and LASA scores at baseline and at 10 weeks were calculated and analyzed with the paired t-test. Any p-value <0.05 was considered statistically significant.

## Results

The median age of 38 participants (5 males, 33 females) was 47 years (range 21-65). All participants completed the 3 instruments used at the beginning and end of the study. Of 380 available massage appointments, 278 were used (mean 7.13, range 1-10 massages per participant). Stress related symptoms improved as follows: the mean PSS-14 score decreased from 17.85 to 14.92 (p=0.0015), and the mean SAS score from 49.45 to 40.95 (p<0.0001). The mean LASA score increased from 42.39 to 44.84 (p=0.0055). Thirty participants (78.95%) felt that their overall job satisfaction improved because of the massages, and 23 (60.53%) were willing to pay $10 to $25 for a 15 minute chair massage if available at work.

## Conclusion

Offering chair massages for nurses in a psychiatric/pain rehabilitation unit during working hours - although difficult to do due to busy clinical schedules - reduced stress related symptoms significantly and was highly appreciated by the nurses.

